# Purchase intention toward sustainable masks after COVID-19: the moderating role of health concern

**DOI:** 10.1186/s40691-022-00317-5

**Published:** 2022-12-15

**Authors:** Sae Eun Lee, Seo Jeong Kim, Kyung Wha Oh, Kyu-Hye Lee

**Affiliations:** 1 Doctoral Research Associate, Human-Tech Convergence Program, Department of Clothing and Textiles, Hanyang University, 222, Wangsimni-ro, Seongdong-gu, Seoul, 04763 Korea; 2 Department of Clothing and Textiles, Hanyang University, 222, Wangsimni-ro, Seongdong-gu, Seoul, 04763 Korea; 3 Professor, Department of Fashion, College of Art, Chung-Ang University, 4726 Seodongdaero, Daeduckmyeon, Anseong, Kyunggi-do, 06974 Korea; 4 Professor, Human-Tech Convergence Program, Department of Clothing and Textiles, Hanyang University, 222, Wangsimni-ro, Seongdong-gu, Seoul, 04763 Korea

**Keywords:** Sustainable mask, Mask pollution, Polymer-based face masks, Health concern, VBN, TPB

## Abstract

This study aimed to investigate consumers’ intentions to purchase sustainable masks to reduce the environmental pollution caused by disposable masks in the context of COVID-19. A research model was derived based on the Value-Belief-Norm theory and the Theory of Planned Behavior, and the moderating role of health concerns and environmental knowledge due to the COVID-19 pandemic were examined. Through a Korean online survey company, we collected data on sustainable masks from respondents aged from their 20 s to 50 s, living in the Korea, and a structural equation analysis was performed on the 337 valid samples. Environmental concerns and beliefs were found to have a positive impact on the purchase intention on sustainable masks. Although environmental knowledge played the role of a moderator, we found that the higher the health concern, the stronger the purchase intention. Based on these results, it is possible to derive a strategy to increase the purchase of sustainable masks and reduce the environmental pollution caused by disposable masks. A sales strategy should be implemented for groups with high health concern. In addition, since the subjective norm increases the purchase intention for sustainable masks, advertising that stimulates them will help reduce environmental pollution caused by disposal masks. In the future, it will be possible to help reduce environmental pollution not only during the COVID-19 pandemic, but also during other emerging pandemics.

## Introduction

With the advent of the COVID-19 pandemic, the world had to face a devastating catastrophe. Due to the strong transmission power, each country took measures up to lockdown to prevent it and issued various action guidelines to prevent the transmission. The World Health Organization (WHO) recommended the use of masks to prevent the spread of the virus (Worby & Chang, 2020). Disposable face masks are playing an important role in preventing the spread of COVID-19 (Citroner, 2022). This has resulted in the rapid production of plastic-based personal protective equipment (PPE). However, the unprecedented rise in the global production of plastic-based surgical or disposable face masks has contributed to increased microplastic pollution (Benson et al., 2021). Discarded plastic masks are broken down into particles smaller than 5 mn, becoming marine plastic pollution, getting absorbed by sea creatures, and finally penetrating into humans as food (Gall & Thompson, 2015). Discarded plastic masks will become microplastic pollution that currently floats, in the amount of 0.39 million tons, in the global ocean (Chowdhury et al., 2021). Therefore, the disposable face masks have produced huge amounts of garbage, which is an emerging problem (Levy, 2022).

Sustainable masks, such as “The Joinery (made from recycled plastic bottles)” “Beta MSK (made from recycled water bottles),” and “Seeus 95(features a natural made of bamboo, silk and carbon)”, are produced to help solve the environmental problems associated with the use of disposable masks (Block, 2020; Gupta et al., 2021; Meisenzahl, 2020). Most previous studies focused on the design of masks for increased performance and convenience (Boix Rodríguez et al., 2021; Venesoja et al., 2021). However, research on the opinion of consumers regarding sustainable masks, remains scarce. Consumers are the actors who can reduce the use of disposable masks and, consequently, environmental pollution. Therefore, research on the use of sustainable masks from the perspective of consumers is necessary.

Since the 1980s, theories on sustainable consumption behavior—such as the Norm-Activation Model (NAM) and the Value-Belief-Norm Theory (VBN)—have been continuously developed, and research is being conducted in various fields, including fashion, food, housing, hotels, and travel. Previous studies demonstrated that biospheric and altruistic-based environmental belief (EB), environmental concern (EC), and environmental knowledge (EK) have predictive qualities (Jung et al., 2016; Quoquab et al., 2020; Saari et al., 2021). Furthermore, the norm that one’s behavior can help prevent negative consequences for the environment appears to be a major variable (Han, 2020; Hosta & Zabka, 2021; Pristl et al., 2021). However, the question remains whether these factors have the same effect on the purchase of sustainable masks. As with other sustainable products, sustainable masks must also consider environmental aspects; however, consumers will prioritize protection and health when choosing a sustainable mask. Therefore, this study differs from previous research on various sustainable products. Little is known about consumers’ purchase intention (PI) on sustainable masks.

To fill this gap in the literature, this study analyzes consumers’ PIs for sustainable masks, using EC and EB as independent variables influencing the purchase of sustainable products. We also examine the effects of subjective norm (SN), i.e. the perceived influence of others and groups. In addition, since we examine COVID-19 masks, we consider health concerns (HC) as an important moderator.

The results of this study are expected to have several theoretical and practical implications. First, this study provides a theoretical basis for the establishment of a sustainable mask as a product that has a crucial impact on personal health. Second, the concept of protection is crucial for a mask; accordingly, it provides focus points for developing future marketing strategies for consumers. Third, promoting sustainable mask purchases could help reduce the environmental pollution caused by disposable masks.

Due to the sudden and catastrophic spread of COVID-19 and the chaos, consumers focused on protection, rather than sustainability. However, it is necessary to think about serious environmental problems caused by the disposal mask and the adverse effects on humans in the end. Based on the research on sustainable masks, we aim to help increase the sustainability of similar plastic-based products (disposable gloves, disposable medical gown etc.) This is because such a pandemic is highly likely to occur repeatedly in the future. This will ultimately help reduce environmental pollution and improve human well-being and sustainability for future generations.

## Theoretical background and hypotheses

### Sustainable mask

A broadly and commonly accepted definition of sustainable consumption posits that sustainable consumption is “the use of services and related products, which respond to basic need and bring a better quality of life while minimizing the use of natural resources and toxic materials as well as the emissions of waste and pollutants over the life cycle of the service or products so as not to jeopardize the needs of future generations” (United Nations Environment Programme [UNEP], 2015). The Lowell Center for Sustainable Production(LCSP)'s (n.d.) (1998) definition of sustainable production is the most widely used: “the creation of goods and services using processes and systems that are non-polluting; conserving of energy and natural resources; economically viable; safe and healthful for employees, communities and consumers; and socially and creatively rewarding for all working people.” Both consumer and producer sustainability emphasize the importance of conserving resources and reducing environmental pollution. Therefore, a sustainable mask can be defined as protecting the wearer from viruses while minimizing the use of natural resources and toxic material during the production stage and minimizing environmental pollution for future generations even after disposal.

The masks currently used against COVID-19 can be divided into four types (Ji et al., 2020): Homemade masks, Reusable masks, Antivirus masks and Degradable masks (Table 1). Considering resource circulation and environmental aspects, all masks, except for the antivirus one, are included in sustainable consumption. However, disposable masks, rather than sustainable ones, are the most used, thus contributing to environmental pollution.

**Table 1:** Classification of COVID-19 masks

Types	Pros	Cons
Homemade masks	Low cost, reusability	Unstable protection
Reusable masks	Reusability	Product development
Antivirus masks	Strong protection	Disposable, pollution
Degradable masks	Sustainability	Expensive

Due to the COVID-19 pandemic, more and more masks were used to prevent transmission, which resulted in an increase in mask waste and, as a new serious environmental problem, this increase in mask waste started to appear as a factor that directly adversely affects human health (Patrício Silva et al., 2020; Ray et al., 2022). In 2021, Proceedings of the National Academy of Science s(PNAS) reported that there were approximately 25,900 tons of plastic COVID-19 waste by face mask and gloves (Radford, 2022), Chowdhury et al. (2021) argued that discarded plastic mask microplastic pollution can amount to approximately 0.39 million tons. Previous studies analyzed this issue from various perspectives. For instance, Selvaranjan et al. (2021) and Hartanto and Mayasari (2021) proposed a synthetic mask with natural plant fiber, upcycling the mask waste, and so forth to reduce environmental pollution due to the increased use of the disposal mask. In another study, Tirkolaee et al. (2022) studied the design a sustainable mask Closed-Loop Supply Chain Network (CLSCN). This was a proposal to minimize total pollution and human risk from locational to production, recycling and disposal decision. Furthermore, research on alternative materials (Tcharkhtchi et al., 2021; Wang et al., 2022; Zhao et al., 2020) or perspective of utility (Howard et al., 2020; Yang et al., 2020) for disposal masks is currently underway. However, research on how to solve this problem form a consumer’s point of view is scare. Hartanto and Triastianti (2022) explored consumers’ eco-friendly mask preference and suggested government policies for face mask waste; overall, most previous studies focused on material, management, and efficacy. In this context, it is necessary to look at how consumers should act, how to reduce consumption of disposal masks, and how to increase consumption of sustainable masks. This is because the main body of consumption is the consumer.

### Value-Belief-Norm theory and Theory of Planned Behavior

Stern’s (2000) Value-Belief-Norm (VBN) theory is significant in sustainable consumption, as it explains pro-environmental behavior. Based on Schwartz’s (1977) Norm-Activation Model of sacrificing oneself for altruistic behavior, it combines Dunlap et al.’s (2000) New Ecological Paradigm. New Ecological paradigm aims to understand how environmentally friendly people are, and it is a measure that helps predict environmental conscious consumption behavior through people’s attitudes and interest in the natural environment. According to the VBN, the process of expressing personal norms, as described in the Norm-Activation Model, is part of the model. Stern (2000) tried to identify human intentions for environmentally friendly behaviors by combining the values, beliefs, and norms of several existing theories. The VBN comprises biospheric, altruistic, and egocentric beliefs, combined with a new ecological paradigm, awareness of the consequences, and perceived ability to reduce threats. Personal norms ultimately influence pro-environmental behavior. Consequently, there has been extensive previous research on sustainable consumption, including sustainable clothing (Carfora et al., 2021), organic food (Chen, 2020), water-saving behaviors (Su et al., 2021), and cosmetic products (Jaini et al., 2019).

The Theory of Planned Behavior (TPB), which explains consumer behavior, uses an approach different from that of the VBN. TPB has been successfully adopted in previous studies related to pro-environment behavior and explains the incomplete volitional control of the Theory of Reasoned Action (Ajzen & Fishbein, 1980). The TPB consists of attitude, SN, and perceived behavioral control. The more positive are the three aspects, the stronger is the behavioral intention (Ajzen, 1991). Various studies on sustainable consumption have been conducted based on the TPB. For instance, Park and Ha’s study (2014) effectively explained recycling behavior, which was later confirmed through studies on sustainable food consumption, apparel, and energy (Alam et al., 2020; Kumar & Mohan, 2021).

Accordingly, based on the above-mentioned theories, we propose a theoretical model with EB and EC in the VBN, and SN, attitude, and intention in the TPB, that can be used to examine sustainable mask use behavior.

## Conceptual development

### Environmental belief and concern

People form beliefs through direct experiences and information, all of which influence their behavior. EB is defined as the perspective of an individual who continuously perceives and recognizes environmental problems (Stern, 2000). Such consumers believe that their efforts to purchase sustainable products create positive changes in relation to environmental issues; they continuously strive to practice EB in action (Pieters et al., 1998). Research finds significant correlation between EB and consumer behavior. In studies of apparel (Lang & Wei, 2019), and biofuels (Pagiaslis & Krontalis, 2014), EB was found to have a positive effect on sustainable consumption behavior. Therefore, consumers using sustainable masks are likely to be aware of environmental pollution; that is, consumers with a high interest in the environment will have strong EBs. In addition, various studies on sustainability have confirmed the predictive qualities of EB.

Fransson and Gärling (1999) defined EC as “ranging from a specific attitude towards environmentally relevant behavior to a more encompassing value orientation.” According to Vainio and Paloniemi (2014), EC refers to the overall value orientation towards the environment, which includes a level of concern about harming the environment due to human progress and the future of the environment. Consumers who have concerns about environmental issues tend to support sustainable products (non-chlorine-bleached paper products, café direct coffee, and green car, etc.) that help to avoid environmental destruction and resource depletion (McDonald & Oates, 2006). Studies on green energy brands (Hartmann & Apaolaza-Ibáñez, 2012), and biofuels (Pagiaslis & Krontalis, 2014) have similarly shown that EC increases pro-environmental behavior.

### Environmental attitude and subjective norm

A definition of attitude is “a psychological tendency that is expressed by evaluating a particular entity with some degree of favor or disfavor” (Eagly & Chaiken, 1993). An environmental attitude (EA) implies that the individual demonstrates value evaluation and reacts to the importance of the environment, taking actions in its interest, and demonstrating pro-environmental consumption behavior. An EA is related to pro-environmental intentions and participation in the purchase, use, and disposal of pro-environmental products (Henninger et al., 2021; Sheoran & Kumar, 2021). Accordingly, EA was analyzed as a major factor in most studies related to pro-environmental consumption (Carfora et al., 2021; Casaló et al., 2019; Ertz et al., 2016; Wyss et al., 2022).

A norm is a certain pattern of behavior constrained and compelled to conform to the society’s unstated rules with a sense of moral responsibility and duty (Schwartz, 1977). In Schwartz’s (1977) Norm-Activation Model, moral is a defined responsibility for the negative consequences of not acting environmentally and plays a key role in converting to environmentally friendly behavior. Previous studies suggested that the norm is a crucial motivating factor affecting pro-environmental behavior, inducing a sense of moral obligation to the environment (Gkargkavouzi et al., 2019; Minton et al., 2018). In the Norm-Activation Model, it appeared in the personal norm research model; however, in the eco-friendly TBP study, the explanatory power was higher, so the SN was adopted in this study (Alam et al., 2020; Kumar & Mohan, 2021; Park & Ha, 2014).

Additionally, previous studies revealed that a high level of EC increases SN for taking pro-environmental actions. For instance, Davari and Strutton (2014) identified a positive relationship between EBs and SNs and the EA of consumers towards sustainable behaviors. Sharma et al. (2017) revealed a positive relationship between EBs and an environmentally-oriented purchasing behavior. Similarly, Lang and Wei (2019) pointed out the positive influence of EBs on the intention to purchase transformable apparel; finally, Nam et al. (2017) determined a positive relationship between attitude and PI towards green sportswear.

Considering the theoretical discussion above, we posit the following hypotheses on the relationship between EB, EC, EA, and SN in sustainable mask use:

H1.EB is positively related to (a) attitude toward sustainable masks and (b) SNs concerning sustainable consumption.

H2.EC is positively related to (a) attitude toward sustainable masks and (b) SNs regarding sustainable consumption.

H3: EA toward purchasing sustainable masks positively relates to a consumer’s PI for sustainable masks.

H4: SN toward purchasing sustainable masks positively relates to a consumer’s PI for sustainable masks.

### Environmental knowledge

Consumer knowledge is related to experience, and since it is stored in an individual’s memory and works when there is an external stimulus, it becomes a variable affecting the cognitive process of searching for and processing information about a given product (Bettman & Park, 1980). EK is defined a person’s ability to identify or define a number of symbols, concepts and behaviors patterns related to environment protection(Laroche et al., 2001), or the concepts and ideas that individuals have based on experiences and a basic understanding of the overall environment and environmental issues (Ellen et al., 1991). In most cases, the higher the EK, the more positive the attitudes toward eco-friendly brands or products, which in turn leads to environmentally conscious behavior (Costa et al., 2021; Wang et al., 2020). In a hybrid vehicle study, Hamzah and Tanwir (2021) found that EK had a significant moderating effect between green PI and perceived green value, but not on SN and attitude. Furthermore, Zheng and Chi (2015) asserted that higher levels of EK strengthen the relationship between consumers’ attitudes and PI. Chi et al. (2019) found that EK positively moderates the relationship between US consumers’ attitudes and their PIs toward sustainable cotton-made collegiate apparel. Considering this, we posit:

H5.EK positively moderates the relationship between consumers’ attitudes toward sustainable masks (a), SN (b), and their PI for sustainable masks.

### Health concern

In order to further protect themselves from the transmission of COVID-19 and to strengthen immunity, consumers started to show interest in various issues to better protect their health. Health concern (HC) refers to the degree of concern about one’s personal health (Gould, 1990). The higher a consumer’s HC, the more effort they put in maintaining and improving their health or quality of life (Spence et al., 2005). Previous research found that pro-environmental consumers exhibit health-conscious behaviors (Hoek et al., 2017). HC appears to be an important factor influencing the PI in organic food, green hotels, and environmental furniture research (Sadiq et al., 2022; Trang et al., 2019; Xu et al., 2020). Additionally, Verma et al. (2019) established that well-being and health are likely to motivate consumers to adopt eco-friendly products. Similarly, Robbins and Wiechelt (2004) suggested that self-care behavior and environmental attitudes are significantly correlated. The higher is the interest in health, the higher is the motivation to just improve one’s health status, even if it is pro-environmental behavior. In the present study, we predict that HC will play an important role because of the pandemic, a situation that is more health-related than that situation examined in previous studies. As most of the news reports suggest using a disposal mask with a strong protective function, such as N95, KF95, to protect the contagiousness of COVID-19 (Cleveland Clinic, 2022), consumers are more likely to use a disposal mask to protect their health than a sustainable mask will use it. Therefore, consumers with high HC would value health protection, despite being aware of the importance of the environment. Furthermore, since such consumers are more likely to be selfish or egoistic (Sadiq et al., 2022), they would moderate the relationship between SN and PI.

H6: HC negatively moderates the relationship between consumers’ attitudes toward sustainable masks (a), SN (b), and their PI for sustainable masks.

## Methods

### Data collection

The questionnaires adopted in this study were distributed through Embrain, an online survey company in South Korea. In the questionnaire, the participants were asked if they had ever used a sustainable mask while describing it. Sustainable masks are explained as multi-use masks that can reused or made from nature-friendly materials, and additionally, masks that develop new materials such as recycling and copper fibers were described. It was set to drop out of the response if there was no experience of using it. Afterwards, the respondents were asked to answer questions while recalling the sustainable mask they used. A total of 350 questionnaires were collected, and after excluding the invalid ones, such as incomplete questionnaires, 337 were used for the analysis. SPSS 22.0 was used for frequency and reliability analysis, and AMOS 22.0 for the measurement model and structural equation model analysis. The demographic characteristics of the participants are as follows: gender was male (49.3%, n = 166) or female (50.7%, n = 171), age was 20’s (25.5%, n = 86), 30’s(24.6%, n = 83), 40’s(24.9%, n = 84), 50’s(24.9%, n = 84), location was urban(59.6%, n = 201), and rural(40.3%, n = 136). Educations was high school (12.8%, n = 43), college (73.6%, n = 248), graduate school (13.6%, n = 46), single (43.9%, n = 148), married (53.4%, n = 180), and others (2.7%, n = 9). The number of families was 1(13.1%, n = 44), 2(12.5%, n = 42), 3(26.1%, n = 88), 4(39.8%, 134), 5 or more (8.6%, n = 29). The experience of using a sustainable mask is that it has been used in the past but is not currently used (42.7%, n = 144), and is currently used (57.3%, n = 193). Average monthly spending on mask purchase was either under $50 (68.5%, n = 231), $50 ~ 100 (24.3%, n = 82), $100 ~ 150 (3.9%, n = 13), and other (3.3%, n = 11).

### Measurement of variables

#### Environmental behavior (EB)

The EB scale was developed following Ellen et al. (1991) and Rothenberg and Matthews (2017). The scale comprised four items, each measured on a 5-point rating scale (1 = strongly disagree, 5 = strongly agree). The reliability of the measure was checked by examining the value of Cronbach’s alpha, which was 0.826.

#### Environmental concern (EC)

The EC scale was developed following a study by Pagiaslis and Krontalis (2014). It comprised five items, each measured on a 5-point rating scale (1 = not at all concerned, 5 = very concerned). The reliability of the measure was checked by examining the value of Cronbach’s alpha, which was 0.855.

#### Environmental attitude (EA)

The attitude scale was developed based on Taylor and Todd’s (1995), and Zheng and Chi’s (2015) research. Attitude was systematized through three items, each measured on a 5-point rating scale (1 = strongly disagree, 5 = strongly agree). The reliability of the measure was checked by examining the value of Cronbach’s alpha, which was 0.883.

#### Subjective norm (SN)

The SN scale was developed following the studies of Chen and Hsieh (2012) and Fitzmaurice (2005). SN was structured through four items, measured on a 5-point rating scale (1 = strongly disagree, 5 = strongly agree). The reliability of the measure was evaluated by examining the value of Cronbach’s alpha, which was 0.891.

#### Purchase intention (PI)

The PI scale was developed following Madden et al. (1992) and Taylor and Todd (1995). PI was systematized through four items, each measured on a 5-point rating scale (1 = strongly disagree, 5 = strongly agree. The reliability of the measure was checked by examining the value of Cronbach’s alpha, which was 0.864.

#### Environmental knowledge (EK)

The EK Scale was developed following Pagiaslis and Krontalis’ research (2014). It comprised three items, each measured on a 5-point rating scale (1 = not at all knowledgeable, 5 = very knowledgeable). The reliability of the measure was evaluated by examining the value of Cronbach’s alpha, which was 0.866.

#### Health concern (HC)

The HC scale was developed based on the research of Dutta-Bergman (2006), and comprised five items, each measured on a 5-point rating scale (1 = strongly disagree, 5 = strongly agree). The reliability of the measure was examined through the value of Cronbach’s alpha, which was 0.836.

## Results

### Validity of the measurement model

Prior to hypothesis testing, confirmatory factor analysis (CFA) was performed to verify the fit and construct validity of the measurement items. CFA was performed using 19 indicators. The results of the CFA were χ^2^ = 386.546 (df = 188), χ^2^/df = 2.056, p = 0.000, NFI = 0.921, GFI = 0.907, CFI = 0.957, and RMSEA = 0.056, showing good fit. The results of the CFA are presented in Table 1. Construct validity was evaluated using convergent and discriminant validity. For convergent validity, the factor loading should be statistically significant with individual values over 0.629, while the average variance extracted (AVE) should be over 0.50; the construct reliability (CR) should be over 0.70. In this study, all of the standard factor loadings were significant and were found to be over 0.60, with AVE over 0.50, and CR over 0.70, confirming convergent validity (Table 2). Additionally, by comparing the squared correlation coefficient and the AVE of each construct, the squared correlation coefficient was found to be lower than the AVE; thus, discriminant validity was also confirmed (Table 3).

**Table 2:** Results of measurement model

Construct	Items	Standardized Factor Loading	t-value	Cronbach’s α	AVE	CR
EC	How concerned are you about the environment? How concerned are you about pollution? How concerned are you about water and air pollution in your city? How concerned are you about extravagant water usage in your city?	0.830 0.895 0.758 0.629	– 19.103*** 15.454*** 12.219***	0.855	0.615	0.863
EB	I am willing to participate in preserving the environment I believe personal responsibility for environmental problems is important I believe the moral obligation to help the environment is important	0.735 0.812 0.811	– 13.653*** 13.500***	0.826	0.620	0.830
EA	Purchasing sustainable masks is a good idea I like the idea of purchasing sustainable masks I have a favorable attitude toward purchasing sustainable masks	0.837 0.868 0.840	– 19.159*** 18.243***	0.883	0.720	0.885
SN	Most people who are important to me think it is a good idea for me to buy sustainable masks Important people in my life want me to purchase sustainable masks People whose opinions I value would prefer that I purchase sustainable masks	0.837 0.878 0.853	18.508*** 19.668*** –	0.891	0.733	0.892
PI	I intend to buy sustainable masks in the future I will try to buy sustainable masks in the future I definitely want to purchase sustainable masks in near future	0.837 0.870 0.785	– 18.937*** 16.437***	0.864	0.691	0.871
EK	How knowledgeable are you about the issue of masks production from renewable materials? How knowledgeable are you regarding the production of masks? How knowledgeable are you regarding different face masks?	0.866 0.854 0.762	– 17.263*** 15.463***	0.866	0.687	0.868
HC	Living life in best possible health is very important to me Eating right, exercising, and taking preventive measures will keep me healthy for life My health depends on how well I take care of myself	0.757 0.875 0.804	– 14.969*** 14.305***	0.849	0.662	0.854

*EC*  environmental concern, *EB* environmental belief, *EA* environmental attitude, *SN* subjective norm, *PI* purchase intention, *EK* environmental knowledge, *HC* health concern

p < 0.001

**Table 3:** The squared correlations and AVE of variables

	EC	EB	EA	SN	PI	EK	HC
EC	**0.615**^**a**^						
EB	0.507^b^	**0.620**					
EA	0.248	0.287	**0.720**				
SN	0.144	0.141	0.452	**0.733**			
PI	0.203	0.263	0.204	0.468	**0.691**		
EK	0.121	0.033	0.066	0.188	0.073	**0.687**	
HC	0.216	0.311	0.161	0.051	0.133	0.025	**0.662**

*EC* environmental concern, *EB* environmental belief, *EA*  environmental attitude, *SN* subjective norm, *PI* purchase intention, *EK* environmental knowledge, *HC* health concern

^a^Average Variance Extracted (AVE) for constructs are displayed on the diagonal and bold

^b^Numbers below the diagonal are squared correlation estimations of two variables

### Hypothesis test

**Fig. 1:**
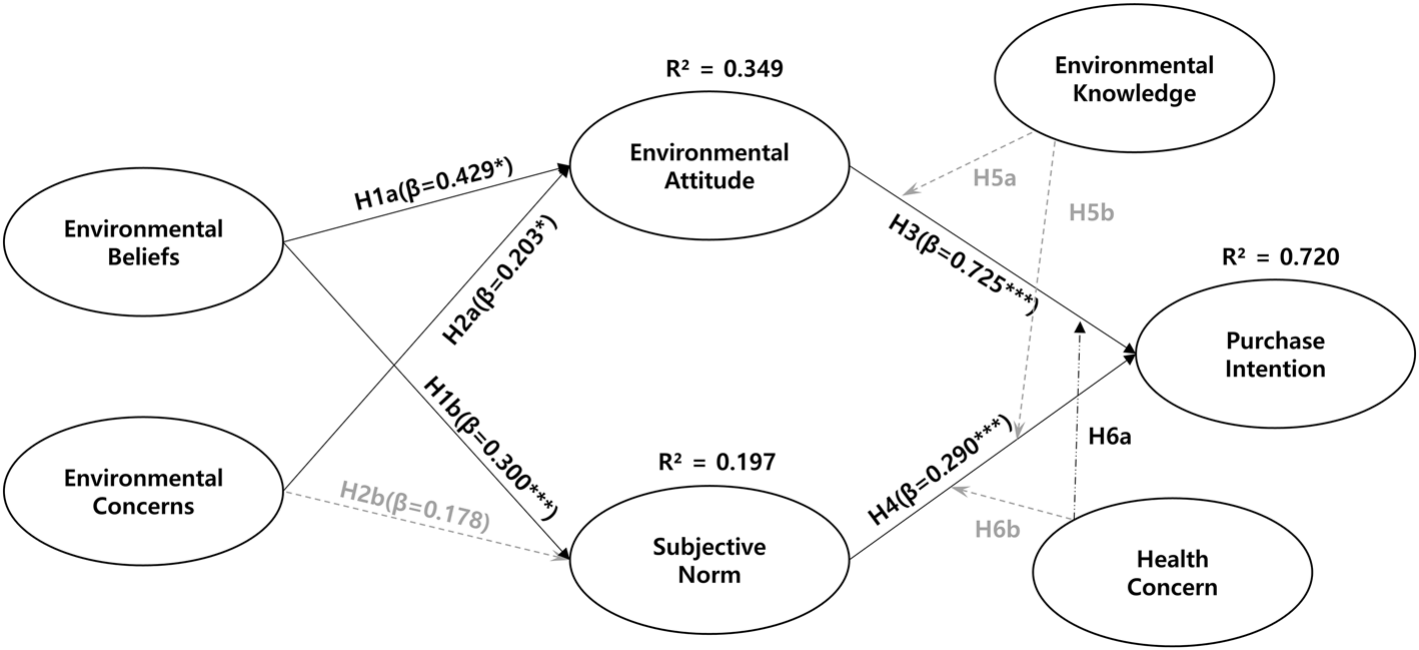
Research model of results. ***p < 0.001, **p < 0.01, *p < 0.05. The model fit indices: χ^2^ = 299.724 (df = 97), χ.^2^/df = 3.090, p = 0.000, GFI = 0.900, NFI = 0.917, CFI = 0.942, RMSEA = 0.079

To test the hypotheses, a structural equation model (SEM) was constructed using the validated items. The results of which are shown in Fig. 1. The structural model fit was χ^2^ = 299.724 (df = 97), χ^2^/df = 3.090, p = 0.000, GFI = 0.900, NFI = 0.917, CFI = 0.942, and RMSEA = 0.079, showing satisfactory goodness of fit. The SEM results showed that EB had a positive effect on EA (β = 0.429, p < 0.000) and SN (β = 0.300, p < 0.01), thus supporting H1a and H1b. EC had a positive effect on EA (β = 0.230, p < 0.05), supporting H2a. However, EC did not affect SN (β = 0.178, p > 0.05); thus, H2b was rejected. EA (β = 0.725, p < 0.000) and SN (β = 0.290, p < 0.000) had a positive effect on PI, thereby supporting H4 and H5. The results of the hypothesis testing are presented in Table 4.

**Table 4:** Result of structural equation modeling and model fit

	Hypothesis	Standardized Coefficient	S.E	*t-*value	Results
H1a	EB EA	0.429	0.095	4.763***	Accepted
H1b	EB SN	0.300	0.128	3.162**	Accepted
H2a	EC EA	0.203	0.077	2.384*	Accepted
H2b	EC SN	0.178	0.106	1.942	Rejected
H3	EA PI	0.725	0.063	12.254***	Accepted
H4	SN PI	0.290	0.039	6.308***	Accepted

*EC* environmental concern, *EB* environmental belief, *EA* environmental attitude, *SN* subjective norm, *PI* purchase intention

***p < 0.001, **p < 0.01, *p < 0.05;

## Moderating effect of EK and HC

To test the moderating effect of EK and HC, a multigroup SEM analysis was conducted. Based on the ratings of EK and HC, respondents were divided into subgroups regarding EK: low (N = 179) or high (N = 158), and HC: low (N = 140) or high (N = 197). The two groups significantly differed in their ratings (for EK: M_high_ = 3.84 vs M_low_ = 2.54, p < 0.000; and for HC: M_high_ = 4.33 vs M_low_ = 3.43; p < 0.000).

Before analyzing the difference between the two groups, a measurement invariance test was performed to confirm the equivalence of the measurement model for its variables (Byrne, 2001). A configurational invariance test was conducted to verify whether the measurement structures between the two groups were the same. The chi-square value was found to be significantly high for the measurement model (EK: χ^2^ = 333.473, df = 188, p < 0.000; HC: χ^2^ = 326.237, df = 188, p < 0.000), and yielded an adequate fit of the model to the data (EK: χ^2^/df = 1.744, GFI = 0.891, NFI = 0.907, CFI = 0.957, RMSEA = 0.048; HC: χ^2^/df = 1.735, GFI = 0.892, NFI = 0.906, CFI = 0.957, RMSEA = 0.047). Thus, configurational invariance between the low and high groups was supported.

The results of the chi-square difference test between the unconstrained model and the measurement weight model were Δχ^2^ = 13.259, df = 11, p = 0.277 (EK), and Δχ^2^ = 17.254, df = 11, p = 0.101 (HC). The non-significant change in model fit showed that the factor loadings were invariant between the two groups, confirming the full measurement-invariance model. For EK with an EA-PI link significant for the high (β = 0.738, p < 0.000) and low (β = 0.706, p < 0.000) groups, no significant differences in path strength were detected by EK (P = 0.653). The effect of SN on PI was significant for the high (β = 0.214, p < 0.000) and low (β = 0.242, p < 0.000) groups, with no significant differences in path strength by EK (P = 0.978). For HC with an EA-PI link significant for the high (β = 0.806, p < 0.000) and low (β = 0.581, p < 0.000) groups, those with high HC (Δχ^2^ = 5.294, P < 0.000) were significantly stronger. The effect of SN on PI was significant for the high (β = 0.225, p < 0.000) and low (β = 0.359, p < 0.000) groups, with no significant differences in path strength by HC (Δχ^2^ = 2.895, P = 0.089). Thus, although the moderator’s role was rejected, H6 was found to be moderate, contrary to the hypothesis of this study (Table 5).

**Table 5:** Comparison of multi-group SEM analysis between two HC and EK groups

Moderator	Path	Std. Estimate		Δχ^2^	Δχ^2^/*df*	*p*
		Low	High			
HC	EA PI	0.581***	0.806***	5.294	1	0.000***
	SN PI	0.359***	0.225***	2.895	1	0.089
EK	EA PI	0.706***	0.738***	0.202	1	0.653
	SN PI	0.242***	0.214***	0.001	1	0.978

*EC* environmental concern, *EB* environmental belief, *EA* environmental attitude, *SN* subjective norm, *PI* purchase intention, *EK*  environmental knowledge, *HC*  health concern

***p < 0.001

## Discussion

This study examined consumer’s purchase intention of sustainable masks to mitigate the problem of environmental pollution caused by disposable masks. The study found that the EC and EB of sustainable masks positively affected PI through consumers’ EA and SN. However, EC had no effect on SN, suggesting that consumers’ EC and EB play an important role in the PI of sustainable masks; this is consistent with the findings of previous pro-environmental consumption studies (Lang & Wei, 2019; Pagiaslis & Krontalis, 2014). The fact that the EC does not affect SN reveals that concerns about the environment should increase the PI of sustainable masks, regardless of people’s opinions and beliefs. Furthermore, EA had a greater influence on PI than SN, implying that when purchasing a sustainable mask, the attitude one has toward the environment is more important than the influence of the surroundings.

The results showed that EK has no moderating effect, which is in contrast to the findings of previous studies (Chi et al., 2019; Hamzah & Tanwir, 2021). EK had the same moderating effect, regardless of whether there was a lot of knowledge about the environment when purchasing a sustainable mask. Regardless of EK, EC and EB have a very strong influence on sustainable PIs. However, the moderating effect of HC was contrary to the proposed hypothesis (H6). HC was found to reversely moderate EA to PI, but not SN to PI. Unlike other sustainable products, the mask specifically protects against infection, which is why the moderating effect of HC was examined, finding that higher HC correlates to stronger PI. This result contradicts that of previous studies (Sadiq et al., 2022; Xu et al., 2020), and it is understood that sustainable masks, which have weaker protective functions than disposable ones, are perceived to be less harmful to their body. In the study of Sadiq et al. (2022), environmental concern is also important; however, health concern had a very important effect on eco-friendly hotel selection. Since this is an eco-friendly hotel, the reason for judged that there will be no harm to one’s body is more important than thinking about the environment. Xu et al. (2020) asserted that environmental consciousness has no significant effect, while health concern had a positive effect on consumers’ intention to purchase authentic green furniture. Although consumers choose sustainable products for their health, we found that consumers who value their health are likely to choose a sustainable mask, rather than a disposable one that likely provides better protection. This is thought to be caused by situational specificity, which differs from the findings of previous studies (Sadiq et al., 2022; Xu et al., 2020).

Theoretical implications are as follows. This study is the first to examine consumers’ PIs for sustainable masks. Whereas studies on sustainable consumption have been conducted, research on masks, especially in the context of COVID-19, remains limited. Existing studies have examined mask design, performance improvement, and production (Boix Rodríguez et al., 2021; Venesoja et al., 2021), but not consumer choice. Therefore, as the environmental pollution caused by disposable masks is severe, we examined variables that could motivate consumers to choose sustainable masks, which can help further research. We found that EK had no moderating effect on COVID-19. Previous pre-COVID-19 studies showed that EK has a significant influence and a moderating effect on environmental protection and sustainability. The high EK group is proactive about the environment and is willing to pay for sustainable products (Dang et al., 2021; Pretner et al., 2021). However, in the context of the COVID-19 pandemic, regardless of the EK level, all consumers were likely to purchase a sustainable mask; however, all consumers had a higher EK, which could explain this result. Due to COVID-19 pandemic, consumers can no longer be indifferent toward the environment and sustainability. Therefore, in future, it is necessary to identify if consumers’ EK has changed before proceeding with the research design. Third, the emergence of a moderating effect differs from that of previous sustainable studies on HC. In previous research, since consciousness is based on egoistic values, consumers were found to choose by focusing on their personal benefits, rather than the environment. Since sustainable products are more beneficial to health, customers are likely to purchase them from a selfish point of view, rather than from an environmental perspective. However, in the present study, despite recognizing that a disposal mask is more beneficial to one’s health than a sustainable mask, the purchase intention of a high HC sustainable mask was high. It is thought that PI was high because that the disposable mask is less harmful to the wearer’s body than the protective function from infectious diseases. Therefore, in future studies, it is necessary to more systematically investigate HC in the case of products related to pandemic. Fourth, our results confirmed, once again, that the SN of the TPB can be explained by the COVID-19 context. Previous studies used personal norm, rather than SN, as the main variable (Quoquab et al., 2020). Indeed, Borusiak et al. (2020) showed that SN had no effect, while personal norm had a strong influence. However, since masks prevent the spread of a contagion, the influence of the surrounding people will be very high. Therefore, in the case of consumption research related to contagion, it helped to confirm that it can be explained by the TPB using SN.

Practical implications are as follows. First, ways to reduce the environmental pollution caused by disposable masks were suggested. As the pandemic persists, the use of masks will inevitably increase continuously, and environmental pollution will exponentially rise. However, encouraging consumers to purchase sustainable masks can help reduce environmental pollution. We aim to induce sustainable purchases not by providing information, but through publicity that highlights concerns about the contamination of disposable masks. It is possible to reduce environmental pollution by stimulating EC using publicity and by strategically promoting various types of sustainable masks. Second, this research provides sales strategy implications for companies producing sustainable masks. Consumers with higher HC are more likely to purchase sustainable masks, which can help expand their marketing strategies. In other words, by targeting consumers with high HC, offline stores can strategically display sustainable masks near health products. Additionally, health products can be promoted online as well. Through these various methods, purchase of sustainable masks can be promoted, which will help expand the market share in the future. Third, this study provides implications for consumers concerned about the environment. Such consumers, being aware that the environment is polluted by the use of disposable masks, are informed that various sustainable masks exist, and purchasing them would be sustainable. Since the protection provided by homemade masks varies depending on the material, other reusable or degradable masks can be used to reduce environmental pollution.

Limitations and future research are as follows. There is a lack of research on HC during a pandemic. Previous studies demonstrated that HC includes egoist characteristics and traits of pro-environmental consumption to improve quality of life for one’s own health. However, due to the unique situation of COVID-19, in the present study, the moderating effect was found to be contrary to previous studies that prioritized self-health based in environmentally friendly consumption situations (Robbins & Wiechelt, 2004; Sadiq et al., 2022; Trang et al., 2019; Verma et al., 2019; Xu et al., 2020). Therefore, future studies should conduct a comparative study on the role of HC in a pandemic. There is also a need to examine the relationship between HC and EC in COVID-19 patients. The variable was set to SN. In our research, the participants in the questionnaire were limited to Korean citizens because, as a collectivist culture, Koreans can react more sensitively to SN than those in individualistic cultures. Additionally, according to SN, consumers of individualistic cultures are more likely to choose personal norm rather than a sustainable mask. Therefore, richer implications can be provided with different results, if cultures were compared separately. EK appears to play no role as a moderator. This can be a result of an increase in the EK of all consumers, which differs from previous studies. Therefore, if future research is conducted on COVID-19 awareness, a more convincing study will have to be conducted that will explain why EK has no moderating effect and what kind of effect EK has on COVID-19 awareness. As shown in the theoretical background, there are various types of sustainable masks. However, since consumers lack awareness of various sustainable masks, the government will have to promote the usefulness of sustainable masks for environmental protection, rather than separately implementing marketing strategies for each company. If the present study had been conducted with a homemade mask, reusable mask, and degradable mask groups, different results might have been derived. Therefore, in future research, if sustainable masks are subdivided and researched, it will be possible to establish mask activation and environmental protection strategies.
